# Application of artificial intelligence in the development of *Jamu* “traditional Indonesian medicine” as a more effective drug

**DOI:** 10.3389/frai.2023.1274975

**Published:** 2023-11-02

**Authors:** Tedi Rustandi, Erna Prihandiwati, Fatah Nugroho, Fakhriah Hayati, Nita Afriani, Riza Alfian, Noor Aisyah, Rakhmadhan Niah, Aulia Rahim, Hasbi As-Shiddiq

**Affiliations:** ^1^Department of Pharmacy, School of Health Sciences ISFI, Banjarmasin, Indonesia; ^2^Faculty of Pharmacy, Pancasila University, Jakarta, Indonesia; ^3^Faculty of Veterinary Medicine, Gadjah Mada University, Yogyakarta, Special Region of Yogyakarta, Indonesia; ^4^Bhayangkara Hospital, Banjarmasin, Indonesia; ^5^Faculty of Pharmacy, Ahmad Dahlan University, Yogyakarta, Special Region of Yogyakarta, Indonesia; ^6^Department of Biology, IPB University, Bogor, Indonesia; ^7^Faculty of Pharmacy, Muhammadiyah Banjarmasin University, Banjarmasin, Indonesia

**Keywords:** traditional Indonesian medicine, *Jamu*, artificial intelligence, machine learning, deep learning

## 1. Introduction

Indonesia has a mega biodiversity that includes plants, animals, and microorganisms. Six thousand plant species have been used empirically as natural resources for traditional medicines by nearly 40 million Indonesians (Elfahmi et al., 2014; Erlina et al., 2022; Sanka et al., 2023). *Jamu* is a traditional Indonesian medicine that has not undergone preclinical or clinical trials but has been empirically proven to maintain health and treat disease as it has been used by Indonesian people for centuries (Elfahmi et al., 2014; Laplante, 2016; Ministry of Health, Republik Indonesia, 2022). *Jamu*, which is a part of Javanese medicinal culture, is also recorded in several classic books such as *Serat Centhini, Serat Primbon Reracikan Jampi Jawi*, and *Serat Kawruh*. These could be the references for further development of current standardized herbal medicine.

The traditional Indonesian herbal medicine (*Jamu*) products that are produced from Indonesia's biodiversity are difficult to examine (Sanka et al., 2023). The development of *Jamu* into phytopharmaceuticals or standardized herbal medicine requires a long research phase and high costs with a risk of an extended research time (Brendler, 2021; Chen et al., 2023). Other challenges in the development of traditional medicine into new standardized pharmaceuticals include the need for sophisticated and high-risk procedures with no certainty of a beneficial pharmacological outcome (Zhu, 2020; Vijayan et al., 2022). Furthermore, high research costs for an extended research period will result in high prices, making it less competitive than modern drugs (Osakwe, 2016). Developing a new drug is estimated to take 10–15 years of research and cost ~US$ 2.8 billion, with an 80–90% failure rate when entering the clinical trial phase (Vijayan et al., 2022).

Taxol (Paclitaxel) is an example of a successfully developed bioactive compound derived from herbs. Taxol is a drug used for cancer that was derived from the Pacific yew tree (*Taxus brevifolia*) (Li G. et al., 2022). It was discovered and developed as an anticancer agent in 1960. The discovery of its anticancer potential began with a screening of extracts of the bark of the Pacific yew tree. The discovery of the Taxol component, Paclitaxel, in 1967 highlighted the accomplishment of the screening stage for bioactive compounds with anticancer action (Wani and Horwitz, 2014). The extraction stage was then developed to facilitate the production of Paclitaxel, thereby encouraging the development stage of the extraction process for the bark of the Pacific yew tree. The extraction method achieved satisfactory results in 1980 with the discovery of the taxol precursor extraction method (10-deacetyl-baccatin III). Phase I clinical trials were conducted in 1984 against several types of cancer and industrial-scale production began a few years later (Horwitz, 2023). The lengthy process of each stage of using Taxol as an anticancer agent can hinder the development of drug discovery and development. Faster discovery and development methods of new drugs from natural products need to be applied to answer future challenges.

One current technology that can overcome the challenges of standardized herbal medicine development from *Jamu* is Artificial Intelligence (AI) (Xiaotong et al., 2022). Implementing AI in developing new drugs from *Jamu* has considerable potential (Erlina et al., 2022) by accelerating preliminary data with the help of an existing database so that several initial screening stages can be done more efficiently. Moreover, searching and matching the input data can be more accessible because AI aids selection with its algorithm and then predicts the possible outcome of the previous study.

## 2. Artificial intelligence: preparation, process, and evaluation

AI could play a role in accelerating the development of new drugs by connecting big data on bioactive molecules, pharmacology, metabolomics, and target diseases (Xiaotong et al., 2022). AI application is highly recommended to complement experimental drug discovery, which requires a more extended stage. AI can assist the drug discovery process with high accuracy to avoid failures in drug development. In previous research, many studies that have gone through a long process and cost money did not find bioactives with benefits (Chen et al., 2022). AI can help select many drug candidates from currently available big data with dynamic, heterogeneous, and significant characteristics (Zhu, 2020).

**Figure 1:**
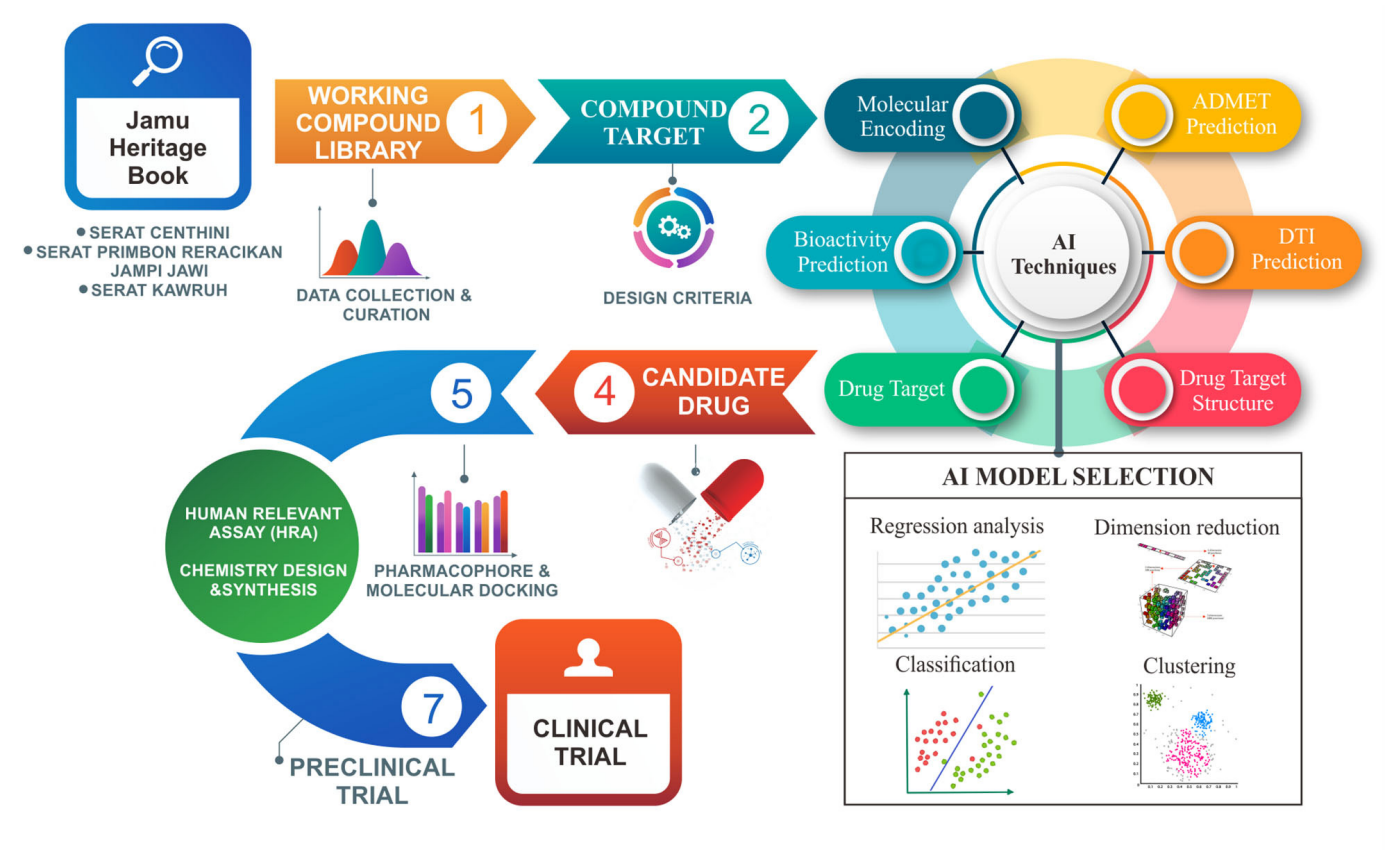
Framework of AI technique application for drug discovery and evaluation.

Drug repurposing for *Jamu* products in Indonesia is the right concept to be combined with AI technology due to several factors, including shortening the 2–14-year research process and utilizing herbal medicine traditionally used by the community (Erlina et al., 2022). The ability of AI to learn from existing data, both data from modern drugs and drugs derived from traditional herbal medicine, makes it possible to obtain new, more effective drugs. Another approach that can be combined is the quantitative structure-activity relationship (QSAR), which is the creation of a model that relates physicochemical properties to biological activity (Zhu, 2020). This system's approach is almost identical to the mechanism approach to drugs and diseases developed to obtain more effective drugs from *Jamu*. The systematic scheme of applying AI in the discovery of new products originating from Traditional Herbal Medicine can be seen in Figure 1 and is described in the following order:

### 2.1. Database identification and data mining: data collection, curation, and compound targets

Databases that can be used and have been tested in several studies include Science Online (http://www.sciencemag.org/), Elsevier Science Direct (https://www.Sciencedirect.com), Springer (https://link.springer.com/), PubMed (https://www.ncbi.nlm.nih.gov/pubmed/), Wiley (https://onlinelibrary.wiley.com/), Nature (https://www.nature.com/), Oxford Academic (https://academic.oup.com/journals/), and Pubchem (https://pubchem.ncbi.nlm.nih.gov/) (Chen et al., 2022; Suttithumsatid et al., 2022). Another database that can be used is the Protein Databank (PDB), an open database with information related to the molecular structure of proteins, nucleic acids, and biological complexes from the results of previous studies (Suttithumsatid et al., 2022). The Indonesian database that contains information on compounds related to *Jamu* is HerbalDB (Erlina et al., 2022). The database has a good data reputation for forming the basis of the big data concept.

A data processing database connects several databases, including ChEMBL (Mendez et al., 2019), ChemDB (Jackson et al., 2020), COCONUT (Sorokina et al., 2021), DGIdb (Freshour et al., 2021), DTC (Li X. et al., 2022), INPUT (Li X. et al., 2022), SIDER (Kuhn et al., 2016), and STITCH (Szklarczyk et al., 2021). The database applies the concept of AI in processing data to produce data that can be used in the development of *Jamu* products. In order to get specific results, the available data need to go through a process called data mining. This process is a data extraction process to obtain valuable data. An AI tool that can be used for data mining is natural language processing (NLP). NLP is a technique in the machine learning (ML) branch that works through sentiment analysis, named entity recognition, machine translation, text summarization, and speech recognition. This technique connects human language in manuscripts with computer language so that data from large databases can get specific results (Saldívar-González et al., 2022).

### 2.2. AI techniques: machine learning and deep learning

Research success in developing new drugs that have therapeutic effects is paramount. Target protein determination must be determined early in development, and bioactive mechanisms may occur. UniProt can be used to determine potential therapeutic targets and signaling pathways involved in the disease being studied (https://www.uniprot.org/). This tool classifies the targets obtained in the database into the following classifications: Druggability, Structure, Diseases, Biology, Genetics, Chemistry, Safety, and Information (De Cesco et al., 2020).

This approach consists of several steps: the first step is a literature study on the database to obtain data on drugs and protein target interactions. The extraction process in the previous stage was to obtain chemical structure and genomic sequence features data. The resulting data is then used as a training dataset. The hyperparameters obtained from the training data set are used to obtain the optimal model. The prediction model for *Jamu* compounds uses data obtained through the previous process (Erlina et al., 2022).

The AI approach uses one of the commonly used branches, including several models tested in previous studies: KNN, SVM, SGD, NB, ANN, Adaboost, LR, MLP, DT, and RF. In addition, the DL model can be combined with the ML model, namely DBN (Chen et al., 2022; Erlina et al., 2022). Through-branch approaches in AI, such as ML and DL, have the same goals but differ in how they work (Vijayan et al., 2022). The ML processes data trained on the program to get a new model prediction. The ML is designed as a model that learns from training data through supervised learning, unsupervised learning, and reinforcement learning (RL). The ML approach to DL uses an artificial neural network (ANN) system to process the data provided and processes the data like a neural network by carrying out learning automatically. The DL approach is suitable for more complex and abstract data but takes longer than the ML approach (Chen et al., 2022, 2023; Vijayan et al., 2022).

Previous research has measured the accuracy of some MLs: 90.1% for RF (Random Forest), 88.2% for MLP (Multilayer Perceptron), 87.4–94.5% for SVM (Support Vector Machine), 84.3% for NB (Naive Bayer), and 78–82.5% for k-NN (k-Nearest Neighbor). These accuracy percentages are not absolute numbers since they can change and depend on the data entered in the ML system, but they can be used as an illustration for selecting the ML system (Kumar and Kumar, 2017; Kaur and Kaur, 2019). The ML is used for preclinical drug development by producing several pieces of information such as on bioactivity, ADMET (Absorption, Distribution, Metabolism, Excretion, and Toxicity), and physicochemical properties (Vijayan et al., 2022). The results of the development of herbal medicine using AI can be seen in Table 1.

**Table 1:** Application of AI to drug development research.

**Artificial intelligence method**	**Natural product**	**Outcome**	**References**
ML	Herbal compounds	31 herbs that have indications as new drug candidates	Kim et al., 2019
ML (random forest)	Traditional Chinese and western medicine combination	The compounds nobiletin, rhein, myricetin, and fisetin have a synergistic effect with celecoxib, and rhein has a positive effect when combined with hydroxychloroquine	Sun et al., 2023
ML (backpropagation artificial neural network)	Jinqi Jiangtang (JQJT)	10 Q-markers have the potential to have bioactivity	Yang et al., 2021
ML (random forest)	Herbal	Procedures suitable for new product development	Esmaeili et al., 2021
ML	Naodesheng (NDS) containing *Crataegus pinnatifida*, Rhizoma Chuanxiong, Radix Notoginseng, *Carthamus tinctorius*, Lobed Kudzuvine	Generate a pharmacological model of the network of constituents found in Naodesheng (NDS)	Pang et al., 2018

The prediction process begins with normalization, and the scaler data is obtained, which is used as the test protein data. The drug data from the database is then merged with the test protein data. This amalgamation is then processed in the ML system in the training data set to obtain the final results of positive and negative interactions. Positive interaction means the drug compound reacts positively with its target protein (Erlina et al., 2022).

Another approach for predicting new compounds in molecules *de novo* obtained from the previous step is the DL system. The currently used DLs are VAEs, generative adversarial networks (GANs), RLs, and RNNs. This approach is also called a generative modeling approach, where AI can produce new models from the training data provided (Vijayan et al., 2022).

### 2.3. Drug candidate: systematic review of proven drugs

Pharmacophore is used to identify critical molecules in the target compounds (bioactive) in *Jamu* against receptors or biological targets. This technique creates a model that is similar to known drug molecules. This technique guides the drug development process to speed up new bioactive compound development (Erlina et al., 2022). Meanwhile, Molecular Docking assesses chemical compounds' interaction with proteins or biological targets in traditional herbal medicine. This modeling method can speed up the development of bioactive compounds that act as ligands at a lower cost than conventional methods (Scior, 2021; Suttithumsatid et al., 2022).

Combining pharmacophore and molecular docking techniques is the final stage in developing new drugs derived from *Jamu*. Pharmacophore modeling can identify critical molecular features, and molecular docking is used to predict the binding of drug molecules obtained with biological targets. After identifying the bioactive structure, the next step is to evaluate ADMET using SwissADME (http://www.swissadme.ch/). SwissADME is a computational-based prediction platform that evaluates drug compounds in terms of their pharmacokinetic and toxicological profiles (Suttithumsatid et al., 2022). ADMET modeling for new compounds can also be done using the ML approach—predictive models developed with neural networks, RF, and SVM systems. The ML used in all three tools is suitable for algorithms with endpoints that have complex and non-linear relationships.

Human-relevant Assays (HRA) is used to observe responses that occur in humans using the *In Silico* method based on molecular data and information relating to biological systems. The final test is Chemical Design and Synthesis to ensure potential drug candidates will be easy to synthesize and produce to meet sustainability aspects (Singh et al., 2023).

## 3. Conclusion

AI can shorten the research process and have a higher success rate for new drug development. The development of new medicines from *Jamu*, which are native Indonesian products and part of the culture of the Indonesian people, can be carried out with AI technology. The stages of drug development from *Jamu* are database identification and data mining, AI Technique, and the systematic review of proven drugs.

## Author contributions

TR: Writing – original draft. EP: Formal analysis, Funding acquisition, Project administration, Resources, Validation, Visualization, Writing – review & editing. FN: Writing – review & editing. FH: Writing – review & editing. NAf: Writing – review & editing. RA: Formal analysis, Funding acquisition, Project administration, Resources, Validation, Visualization, Writing – review & editing. NAi: Formal analysis, Funding acquisition, Project administration, Resources, Validation, Visualization, Writing – review & editing. RN: Conceptualization, data curation, Investigation, Methodology, Software, Supervision, Writing – review & editing. AR: Conceptualization, Data curation, Investigation, Methodology, Software, Supervision, Writing – review & editing. HA-S: Conceptualization, Data curation, Investigation, Methodology, Software, Supervision, Writing – review & editing.
